# Neuronal Oscillations in Various Frequency Bands Differ between Pain and Touch

**DOI:** 10.3389/fnhum.2016.00182

**Published:** 2016-04-29

**Authors:** Georgios Michail, Christian Dresel, Viktor Witkovský, Anne Stankewitz, Enrico Schulz

**Affiliations:** ^1^Department of Neurology, Technische Universität MünchenMunich, Germany; ^2^TUM—Neuroimaging Center, Technische Universität MünchenMunich, Germany; ^3^Neurophysics Group, Department of Neurology, Charité—Universitätsmedizin BerlinBerlin, Germany; ^4^Department of Theoretical Methods, Institute of Measurement Science, Slovak Academy of SciencesBratislava, Slovak Republic; ^5^Oxford Centre for Functional Magnetic Resonance Imaging of the Brain, Nuffield Department of Clinical Neurosciences, University of OxfordOxford, UK

**Keywords:** pain, touch, perception, neuronal oscillations, time-frequency analysis, intensity coding, EEG

## Abstract

Although humans are generally capable of distinguishing single events of pain or touch, recent research suggested that both modalities activate a network of similar brain regions. By contrast, less attention has been paid to which processes uniquely contribute to each modality. The present study investigated the neuronal oscillations that enable a subject to process pain and touch as well as to evaluate the intensity of both modalities by means of Electroencephalography. Nineteen healthy subjects were asked to rate the intensity of each stimulus at single trial level. By computing Linear mixed effects models (LME) encoding of both modalities was explored by relating stimulus intensities to brain responses. While the intensity of single touch trials is encoded only by theta activity, pain perception is encoded by theta, alpha and gamma activity. Beta activity in the tactile domain shows an on/off like characteristic in response to touch which was not observed in the pain domain. Our results enhance recent findings pointing to the contribution of different neuronal oscillations to the processing of nociceptive and tactile stimuli.

## Introduction

The somatosensory system senses environmental stimuli (e.g., mechanical, thermal, vibrational or painful stimuli) by different types of skin receptors. Modality-specific sensory information is then conveyed from the periphery to the cerebral cortex via separate fiber tracts, e.g., the lateral spinothalamic tract for the transmission of pain and temperature (Melzack and Casey, 1968; Dostrovsky and Craig, 2006) as well as the lemniscal pathway for the transmission of fine touch and proprioception (Hall and Guyton, 2011). At cortical level studies have shown differences and similarities for the processing of the somatosensory submodalities pain and touch.

In the past, studies that investigated the pain modality often used somatosensory stimulation only as control condition (e.g., Seminowicz et al., 2004; Segerdahl et al., 2015). Only a few studies so far compared the differences between these two independent sensory systems directly. Some of these studies found a similar cortical activity pattern for nociceptive and tactile stimuli, i.e., the same neurophysiological processes (e.g., alpha and gamma frequencies; Mouraux and Iannetti, 2009; Legrain et al., 2011) in similar brain regions (Mouraux et al., 2011) by activating an equal number of voxels (Taylor and Davis, 2009). Others provided evidence for processes that are specific for pain and touch (Liang et al., 2013; Chien et al., 2014). A functional Magnetic Resonance Imaging (fMRI) study found a distinguishable pattern of activity for pain and touch in primary sensory areas (Liang et al., 2013). By contrast, a further imaging study observed largely overlapping activity between nociceptive and somatosensory responses in the primary somatosensory cortex (Mancini et al., 2012). For neurophysiological research, a more recent electroencephalography (EEG) study by Chien and colleagues (Chien et al., 2014) compared the averaged response to laser pain stimuli and non-painful electrical somatosensory stimuli. While controlling for attentional effects by randomizing the delivery of pain and touch trials, the authors found differences between both stimuli in delta/theta, alpha, beta and gamma frequency bands.

The present EEG study aimed at investigating the differences and commonalities of pain and touch processing in the human brain. Two novel aspects will be explored. First, we will show the topographical distribution of the averaged neuronal responses to laser pain stimuli and touch stimuli. Tactile processing will be explored with an elaborated pneumatic device for delivering natural stimuli that are comparable with laser pain trials in terms of latency and duration. Second, we will investigate and compare—at single trial level—how the encoding of intensities of both modalities is subserved in the human brain. Linear mixed effects models (LME) will quantify which neuronal responses vary with different intensities of pain and touch. We aim at exploring which responses commonly or uniquely encode the intensities of pain and touch.

## Materials and Methods

### Subjects

Nineteen healthy male human subjects with a mean age of 24 years (21–31 years) participated in the experiment, which formed the control condition of a study on the role of dopamine in pain processing (Tiemann et al., 2014). Importantly, the data presented here only include the condition without alteration of the individuals’ dopamine levels. The day prior to testing the subjects received a low protein diet (<10 g of protein; Loprofin^®^ Products, Heilbronn, Germany) and were asked to refrain from the consumption of alcohol, caffeine and analgesics. All subjects gave written informed consent. The study was approved by the local ethics committee and conducted in conformity with the declaration of Helsinki.

### Paradigm

In two consecutive counterbalanced conditions, 75 painful cutaneous laser stimuli and 75 tactile stimuli of matched intensities were delivered to the dorsum of the right hand. The laser device used was a Tm:YAG laser (Starmedtec GmbH, Starnberg, Germany) with a wavelength of 1960 nm, a pulse duration of 1 ms and a spot diameter of 5 mm. The physical energy of the painful stimulation was kept constant at 600 mJ. To prevent skin damage, the stimulation site was changed slightly after each stimulus. Tactile stimuli with a force of 181 mN were applied using von Frey monofilaments delivered through a computer-controlled device as described in detail in Dresel et al. (2008). To incorporate the time lag required for heating up and passively cooling down of the skin during the painful laser stimuli and due to device constraints, a duration of 80 ms for tactile stimulation was used to achieve a comparable subjective stimulus exposure for both modalities. Although tactile transmission (Aβ fibers) from the periphery to the cortex is faster than nociceptive transmission (observed cortical responses mainly related to the activity of Aδ fibers), this effect is counterbalanced by the response delay of the pneumatic device (Dresel et al., 2008). Indeed, the peaks of the N2 and P2 deflections as well as the peak of the subjects’ theta activity at electrode Cz (see “Supplementary Material”) did not exhibit a significant latency difference between pain and touch trials (paired *t*-test, *p* > 0.05).

Interstimulus intervals (ISI) for both modalities were randomly varied between 8 and 12 s. To prevent excessive eye movement related artifacts and blinks the subjects perceived the stimuli with closed eyes. Three seconds after each stimulus, the subjects were prompted by an auditory cue to verbally rate the perceived intensity of the stimulus on a 0–10 numerical rating scale. For pain stimuli this was anchored by no pain (0) and maximum pain (10) the subjects were willing to tolerate during the experiment. For the rating of tactile stimuli, the scale ranged between no perception (0) and maximal imaginable touch (10) that was not perceived as painful.

### EEG Recordings and Analysis

EEG data were recorded using an electrode cap (FMS, Munich, Germany). The electrode montage included 64 electrodes consisting of all 10–20 system electrodes and the additional electrodes Fpz, FCz, CPz, POz, Oz, Iz, AF3/4, F5/6, FC1/2/3/4/5/6, FT7/8/9/10, C1/2/5/6, CP1/2/3/4/5, TP7/8/9/10, P5/6, PO1/2/9/10, plus two electrodes below the outer canthus of each eye. The EEG was referenced to the FCz electrode, grounded at AFz, sampled at 1 kHz with 0.1 μV resolution. Impedance was kept below 20 kΩ.

Raw EEG data were preprocessed in Vision Analyzer Software (Brain Products, Munich, Germany) including downsampling to 512 Hz, high-pass filtering of 0.5 Hz, correcting for horizontal and vertical eye movements using an independent component analysis, and transforming to the common average reference. Sections of EEG that exceeded ±100 μV in any channel were marked as contaminated with artifacts. Artifact-free trials were epoched from −1100 to 1500 ms and exported to Matlab (The Mathworks, Natick, MA, USA). Time-frequency analyses were performed in Matlab using custom programming on the basis of standard mathematical and signal analysis functions. We applied a single trial Hamming tapered, short-time Fast Fourier Transformation (FFT). The moving window had a length of 100 data points, was padded with zeros up to 512 data points and was shifted by two data points. The frequencies were computed from 2 Hz in steps of 2 Hz (interpolated) up to 100 Hz. On a single trial basis, time-frequency representations (TFRs) were computed and transformed into percent signal change values with respect to the single trial baseline averaged from −1000 to 0 ms. These single trial TFRs were visually inspected for high-frequency artifacts (see examples in “Supplementary Material”). For each subject and electrode, the artifact-free and baseline-corrected single trial TFRs were averaged across trials separately for pain and touch.

### Neuronal Responses to Pain and Touch

In a first step, we determined pain-related and touch-related changes of neuronal activity. For each electrode, group TFRs were calculated by averaging the individual TFRs across subjects. Statistical significant changes of neuronal activity were assessed by calculating paired *t*-tests between activity at each data point of the TFRs and the mean activity of the prestimulus baseline period (−1000 to 0 ms) for each electrode. To control for type I error, false discovery rate (FDR) correction across time (0–1000 ms), frequency (2–100 Hz) and electrodes (65) was performed (Benjamini and Hochberg, 1995). To show the topographical distribution of the responses, time-frequency windows were selected based on our previous findings, i.e., theta activity between 4–8 Hz and 150–350 ms, gamma activity between 76–86 Hz and 150–350 ms, alpha activity between 8–16 Hz and 500–700 ms (Schulz et al., 2011). In addition, beta activity was analyzed in the time-frequency window between 16–28 Hz and 600–1000 ms. For the selected responses, neuronal theta activity relates mainly to the phase-locked (evoked) and to a minor extent to non phase-locked (induced) aspects of cortical activity. Alpha, beta and gamma responses reflect the induced cortical activity (Iannetti et al., 2008; Schulz et al., 2011, 2012b). Again, to control for type I error, FDR correction has been applied for all comparisons (4 frequency bands × 65 electrodes).

### The Neuronal Coding of Pain Intensity and Touch Intensity

In a next step, we related the single trial ratings to the single trial neuronal responses of pain and touch. By using the “lmer” function of the statistical software R^1^, we computed LMEs to explore the strength of the relationship between neuronal responses and perception. The expected value of the response variable “rating” is modeled by a linear (regression) function that depends on the explanatory variable brain activity (e.g., theta responses). LMEs were computed separately for each sensory modality and frequency band and took the single trial responses of all electrodes into account. This approach, to compute the LMEs across all electrodes, made it unnecessary to correct for multiple comparisons. The fixed effect of the model reflects the strength of the relationship between a neuronal response and a single percept. The inclusion of random effects (i.e., the independent random intercepts and slopes for each electrode and each subject) allow for modeling a proper covariance structure. A preliminary analysis revealed that the variance components of the random effects (1 | electrode) and (−1 + theta | electrode) were not significantly different from zero. This finding justified the application of a more simplified model (R command exemplarily shown for theta responses to pain) of the form:

$$\begin{array}{l} painmodel<\text{​​}-lmer(painrating\sim theta+\left(1|subj\right)\\ \text{​​​​ }+\left(-1+theta|subj\right),thetapaindata) \end{array}$$

In addition, a further set of LMEs were computed to directly compare the relationships between both modalities, pain and touch (again, across all electrodes but separately for each frequency band). This comparison aimed at elucidating whether pain or touch exhibited a stronger relationship between neuronal responses and perception. Again, the linear function of the model (R command exemplarily shown for theta responses to pain) computes separate parameter values (intercept and slope) for each modality.

$$\begin{array}{l} comparison<\text{​​}-lmer(rating\sim theta+modality\\ \;+theta:modality+(1|subj:modality)\\ \;+(-1+theta|subj:modality),thetadata) \end{array}$$

For each frequency, the resulting *t*-values of the fitted models are related to the estimated coefficient for the fixed effects (for the models within one modality). The interaction coefficients “theta:modality” (for the models comparing both modalities) quantifies whether there’s a difference in the strength of cortical encoding between pain and touch. FDR correction across all frequency band was utilized to correct the computation of four independent models (theta, alpha, beta, gamma band).

## Results

### Behavioral Data

Laser stimuli elicited moderately painful pinprick-like sensations with a mean subjective pain intensity of 3.7 across subjects. Pain ratings elicited by the repeated application of identical stimuli varied considerably within individuals. A root mean square standard deviation of pain ratings within individuals of 1.5 reveals a substantial intraindividual variability in the perception of pain (Lanier, 1943; Boly et al., 2007; Ploner et al., 2010; Schulz et al., 2012b). Tactile stimuli elicited touch sensations with a mean subjective intensity of 3.5. A root mean square standard deviation of 1.2 for tactile ratings also reveals a high trial-by-trial variability. In those subjects that showed a significant difference between mean pain ratings and mean tactile ratings, we stepwise excluded the highest rated trials in one modality and the lowest rated trials in the other modality, until both no longer differed significantly (two-sample *t*-test, *p* > 0.05). This procedure may contribute to compensating for the difference in attention the modalities may draw (Wang et al., 2010; Liang et al., 2013). In addition, as pain and touch exhibit a fundamentally different sensory experience, this matching procedure does not imply that the stimuli were perceived with similar intensities. On average, 50 pain trials (range 26–69) and 56 touch trials (range 35–74) remained after artifact correction and matching procedure (on average 67 and 61 after artifact correction). The number of remaining trials was different between both modalities (mean difference = 6; paired *t*-test, *p* < 0.05) The average of ratings did not change after artifact correction and matching procedure (average of 3.7 for pain and of 3.4 for touch). Due to the possibility, that both types of stimuli may elicit different levels of attention and saliency, we do not directly compare the EEG amplitudes. To explore the different neuronal processing of pain and touch we analyzed the following two aspects.

### Neuronal Responses to Pain and Touch

**Figure 1:**
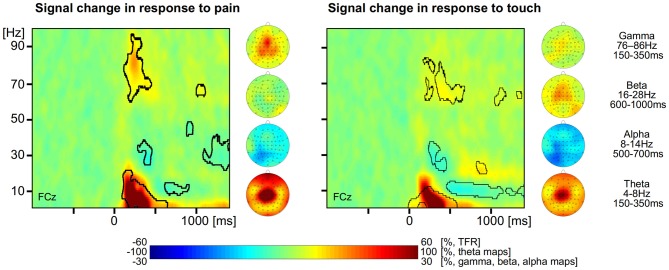
**Neuronal responses to pain and touch stimuli.** Left, time-frequency representation (TFR) and topographical maps of pain-related neuronal activity coded as percent signal change with respect to a prestimulus baseline. Right, TFR and topographical maps of touch-related neuronal activity coded as percent signal change with respect to a prestimulus baseline. Areas with statistically significant responses (*p* < 0.05, false discovery rate (FDR) corrected) are framed by a black line.

For the analysis of EEG data, we first determined neuronal responses to painful and tactile stimuli. TFRs were calculated for each trial and electrode. For the painful stimuli, the group mean TFR at exemplary vertex electrode FCz shows that the brief painful stimuli yielded neuronal responses at latencies between 150 and 1000 ms after stimulus application. We found a strong increase of neuronal activity (210% max. signal change) with a maximum in the theta frequency range (4–8 Hz) at latencies between 150 and 350 ms. The theta response has been shown to be phase-locked to the stimuli and corresponds, hence, to the pain-evoked potential. In addition, theta response and evoked response share the same topographical distribution (Schulz et al., 2011). We also found a less pronounced increase of neuronal activity (28% max. signal change) in the gamma frequency band between 76 and 86 Hz at latencies between 150 and 350 ms and identified a pain-related decrease in neuronal activity (−20% max. signal change) in the alpha range starting at about 500 ms after stimulus application (Figure 1 left, all *p* < 0.05, FDR corrected). There was no significant effect in the beta range. The topographical maps of the different pain-related neuronal responses show that theta and gamma responses are strongest at vertex electrodes whereas the alpha suppression is strongest at left parieto-occipital electrodes.

For the tactile stimuli, the group mean TFR at exemplary electrode FCz shows similar neuronal responses to those in response to painful stimuli (Figure 1 right). We found a strong increase of neuronal activity (186% max. signal change) in the theta frequency range (4–8 Hz, 150–350 ms), a slight increase of neuronal activity (13% max. signal change) in the gamma frequency band (60–80 Hz, 400–600 ms) as well as a decrease (−22% max. negative signal change) of alpha band activity (8–14 Hz, after 500 ms). In addition we also found an increase (26% max. signal change) of beta activity (16–28 Hz) starting at 700 ms after stimulus application (all *p* < 0.05, FDR corrected). The topographical maps of the different neuronal responses to tactile stimuli show that theta, beta and gamma responses are strongest at frontal and vertex electrodes whereas the alpha suppression is strongest at left parieto-occipital electrodes.

### Neuronal Coding of Pain Intensity and Touch Intensity

**Figure 2:**
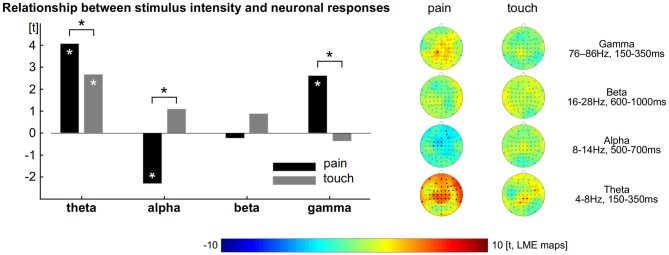
**Relationship between perception and brain responses.** Left: theta, alpha and gamma activity encode the subjective intensity of pain. Theta encodes the subjective intensity of touch. The comparison shows theta and gamma activity as well as alpha suppression to exhibit a tighter connection to pain than to touch. Asterisks indicate statistical significance (*p* < 0.05, FDR corrected). Right: topographical distribution of the relationship between perception and brain activity separately computed for pain and touch.

To explore and to compare the strength of the relationship between neuronal responses and perception we computed LMEs (Figure 2). The intensity of single painful laser stimuli is encoded by neuronal theta (all electrodes: *t* = 4, *t*_max_ = 4.7 at FT10), alpha (all electrodes: *t* = −2.3, *t*_min_ = −3.8) and gamma (all electrodes: *t* = 2.6, *t*_max_ = 3.8 at CP1) oscillations (Schulz et al., 2011). We did not find any significant effect for beta for the encoding of pain (Figure 2 left, all *p* < 0.05, FDR corrected).

For the tactile domain we revealed that the amplitude of single trials is encoded by neuronal theta responses (all electrodes: *t* = 2.7, *t*_max_ = 3.4 at CP2). We did not find any significant effect for alpha, beta and gamma responses for the encoding of touch. The relationships between ratings and neural oscillations in the theta, alpha and gamma range were always stronger for pain than for touch (Figure 2 left, all *p* < 0.05, FDR corrected). To illustrate the distribution of the effects and the contribution of each electrode, we computed further LMEs separately for each modality, electrode and frequency band. The results confirm those obtained from the prior models (topographical maps in Figure 2 right, all *p* < 0.05, FDR corrected).

## Discussion

The aim of the present study was to explore, which neurophysiological responses enable the brain to recognize, differentiate and evaluate the intensity of incoming tactile and nociceptive stimuli. We specifically focused on the following two aspects: first, we analyzed the general neuronal responses to both nociceptive and tactile stimuli. Second, we explored how the intensity of both modalities is encoded in the human brain.

### Neuronal Responses to Pain and Touch

For the processing of painful and tactile stimuli, we found a similar pattern of neuronal responses across scalp electrodes, i.e., an increase of gamma and theta activity as well as a decrease of alpha activity. Increased beta activity was found only in the tactile domain.

#### Theta

Increased theta activity was observed in response to both, pain and touch. Amplitudes of tactile theta responses—particularly at central and parietal electrode sites—appear to be smaller than theta responses to pain. The critical question here is whether the difference in amplitude between both modalities can be attributed to a modality-specific processing or to a modality-unspecific difference in saliency. Higher amplitudes of theta oscillations have been shown to reflect the involuntary attention that novel and salient sensory stimuli are drawing (Iannetti et al., 2008; Wang et al., 2010). However, a recent study found theta differences between painful laser stimuli and electrical non-painful stimuli even after controlling for attentional effects by randomizing the application of both types of stimuli (Chien et al., 2014). This controlling for attention might have been unnecessary as Hauck and colleagues did not find low-frequency activity to be modulated by attention (Hauck et al., 2007, 2015). A further argument that theta activity is not only related to saliency comes from placebo studies which revealed that pain-related theta activity is also affected by placebo interventions (Watson et al., 2007; Lyby et al., 2011). It appears to be unlikely that placebo interventions affect attentional processes. Overall, these findings argue against the exclusive role of theta activity to be related to attention and saliency. Therefore, at least partial aspects of theta activity are considered to be attributed to the differential cortical processing of pain and touch.

#### Alpha and Beta

We further found a stronger decrease of alpha suppression for touch compared to pain as well as a subsequent beta rebound exclusively for tactile responses (Cheyne et al., 2003; Bauer et al., 2006; Dockstader et al., 2008). It can not be unequivocally answered, whether the alpha and beta activity observed here is specific for the tactile domain or is caused by differences in attention. A previous study found unattended somatosensory stimuli to induce an initial alpha and beta suppression and a stronger beta rebound after 500 ms. The study suggests that alpha suppression and beta rebound is inversely related to attention (Bauer et al., 2006). Notably, in the present study, beta oscillations were not present for nociceptive events at all. Overall, these findings suggest that beta responses are considered to play a fundamental role in the somatosensory system (Cheyne et al., 2003; van Ede et al., 2015), after motor responses (Jurkiewicz et al., 2006; Schulz et al., 2012a), but not for nociceptive responses (Hauck et al., 2015; Schulz et al., 2015). Tactile beta oscillations have been suggested to be involved in functional binding processes within somatosensory cortical areas (Simões et al., 2003; Brovelli et al., 2004), in establishing a feed-forward loop to connect somatosensory regions to parietal and frontal brain regions (Adhikari et al., 2014), as well as for differentiating pleasant from unpleasant tactile stimuli (Singh et al., 2014). Beta oscillations over the contralateral somatosensory cortex have further been found to encode information about the spatial position of the index finger (Weichwald et al., 2014). Moreover, beta oscillations might also be involved in inter-hemispheric communications; the amplitude of beta oscillations has been reported to be related to the size of the corpus callosum (Stancák et al., 2003).

#### Gamma

Increased gamma activity was observed in response to both, pain and touch. Amplitudes of tactile gamma responses appear to be smaller than nociceptive gamma responses. Interestingly, touch-related gamma oscillations occurred in a different frequency range (~70 Hz for touch vs. ~80 Hz for pain) and appeared slightly later than pain induced gamma oscillations. This finding suggests that different neuron ensembles—operating in distinct frequencies—contribute to the perception of pain and touch. Previous research has shown separately for pain (Hauck et al., 2007) and touch (Bauer et al., 2006; Karns and Knight, 2009) that attended stimuli exhibit an increase of neuronal gamma activity. Due to the different time windows and frequencies, it appears unlikely that the higher amplitudes of pain-related than touch-related gamma responses could be explained by differences in attention.

#### Pain-Induced Artifacts in the Gamma Range

The pain-related gamma results of the present experiment are in line with previous other findings (Gross et al., 2007; Schulz et al., 2011) but differ substantially from a recently published study on the comparison of pain and touch (Chien et al., 2014). Although the authors of this recent study did not specify any time-frequency windows (Chien et al., 2014), some of the laser-induced neuronal responses occur in an unusually late time window after 500 ms compared to other findings (Schulz et al., 2011, 2012a; Zhang et al., 2012; Tiemann et al., 2014). The late and deviant location of their gamma effects at frontal electrodes (as compared to gamma changes at vertex or lateral electrodes in previous findings) and the present finding might be caused by confounding muscular activity (see “Supplementary Material” and recommendations in Muthukumaraswamy, 2013). The authors did not display any topographical distributions of their effects, which would have made their results more reliable and comparable (Muthukumaraswamy, 2013). It needs to be emphasized that painful stimulation is likely to cause involuntary movements of head muscles in a subset of trials. These movements generate electrical fields that will be picked up by EEG electrodes and potentially contaminate cortical data at frequencies above 30 Hz (Yuval-Greenberg et al., 2008; Shackman et al., 2009). Therefore, it is mandatory to put effort to the detection and removal of artifacts by either extracting data from artifact-free ICA components (Hu et al., 2010), by removing ICA components that represent artifacts, or by excluding an entire trial from subsequent analysis. As these artifacts exhibit higher amplitudes than cortical responses, the careful inspection of single trial decomposed ICA components and EEG epochs is of particular importance. The present study provides the topographical distribution of all effects across the scalp which is mandatory to “separate the wheat from the chaff” (Fries et al., 2008; Muthukumaraswamy, 2013).

### Neuronal Coding of Pain and Touch

In a second step, we related single trial neuronal responses to single trial ratings. To our knowledge, this is the first study that analyzed the relationship between tactile perception and neuro-oscillatory activity in a within-subject design. We found for the tactile domain that low-frequency responses in the theta range encodes for subjective stimulus intensity: theta is positively correlated with touch ratings. Although we revealed a general response of alpha and beta activity in response to all tactile stimuli (see above), these frequencies do not code for the perceived intensity. It seems that alpha/beta activity rather follows an on/off like response characteristic. This could reflect either an involvement in information transmission (or binding), irrespective of different levels of subjective stimulus intensity (Simões et al., 2003; Brovelli et al., 2004; Weichwald et al., 2014), non-specific epiphenomena (Schulz et al., 2011), or an active suppression of brain regions (Neuper et al., 2006; Ploner et al., 2006).

The analysis of the neuronal coding of laser induced brain signals confirmed recent findings about the role of theta, alpha and gamma activity for the processing of pain (Schulz et al., 2011, 2015; Zhang et al., 2012; Hauck et al., 2015). For the present study, these relationships have been shown to be stronger for the pain modality than for the touch modality. While the contribution of theta activity for both modalities cannot be unequivocally answered, alpha and gamma activities (~80 Hz) contribute exclusively to the coding of pain.

### Limitations

Although the ratings were matched for mean and variance, this does not imply that the rating scales for both modalities are directly comparable. Painful stimuli are commonly believed to be more salient than stimuli of any other sensory modality. This might be particularly true for the present paradigm in which the participants were prompted to keep their eyes closed. Therefore, differences in saliency and attention may cause some of the effects presented here. Neuronal responses in the theta and gamma range have been shown previously to be related to salience (Iannetti et al., 2008) and attention (Hauck et al., 2007, 2015). For this reason we do not directly compare the amplitudes of pain and touch responses. These limitations may not apply to the LME analysis in which we compare the *relationship* between cortical activity to pain and pain ratings with the *relationship* between cortical responses to touch and touch ratings. Possible differences for the levels of saliency and attention between pain and touch would be modeled as random effects and not be considered for the statistics of the fixed effects.

Despite the application of physically identical stimuli we found a remarkable variability for the perception of pain and touch. Although we would assume that fluctuating cortical processes play an important role for this variability we also need to consider other sources of variability. These sources that were not systematically controlled for include the fiber density of receptors in different skin areas as well as temperature fluctuations of the hand surface. The LME that quantifies the cortical representation of perceptual variability does not distinguish between the cortical and peripheral sources of variability.

### Summary

Our results demonstrate activity changes in the theta, alpha and gamma range in response to pain. These neuronal responses also encode the intensity of single pain events. For the tactile domain—besides theta, alpha and gamma frequencies—we also revealed increased beta activity in response to all trials. However, the intensity of touch trials was encoded only by theta. Therefore, touch-related alpha and beta responses are suggested to exhibit an on/off like characteristic that is independent from stimulus intensity. The pattern of the present findings, particularly in the alpha, beta and gamma range, suggests that the processing of pain and touch can be attributed to different neuronal ensembles. Further research is needed to investigate the specific contribution of each neuronal oscillation at cellular level, e.g., the mechanisms by which alpha oscillations desynchronize stronger for pain than for touch and encode pain intensity but not touch intensity.

## Author Contributions

All authors listed, have made substantial, direct and intellectual contribution to the work, and approved it for publication.

## Conflict of Interest Statement

The authors declare that the research was conducted in the absence of any commercial or financial relationships that could be construed as a potential conflict of interest.

## Footnotes

^1^http://www.R-project.org
