# Supplementary material for: Neuronal Oscillations in Various Frequency Bands Differ between Pain and Touch
Source: Front Hum Neurosci. 2016 Apr 29;10:182. doi: 10.3389/fnhum.2016.00182 (PMC4850848; doi:10.3389/fnhum.2016.00182)
Supplement: Supplementary file 1 [file Presentation_1.ppt]

## Slide 1
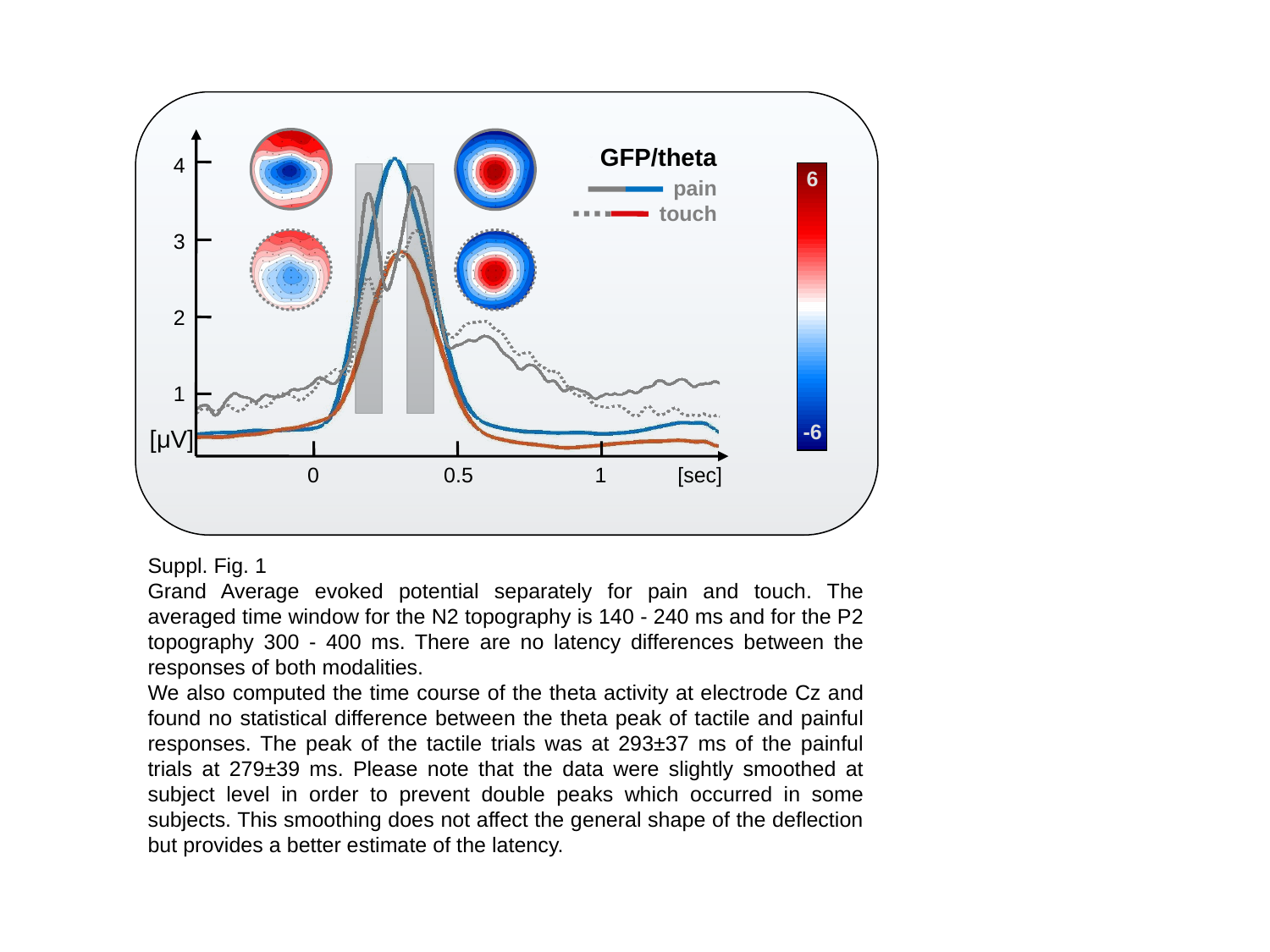

GFP/theta
 pain
touch
4
3
2
1
6
-6
[μV]
0 	 0.5	 1 [sec]
Suppl. Fig. 1
Grand Average evoked potential separately for pain and touch. The averaged time window for the N2 topography is 140 - 240 ms and for the P2 topography 300 - 400 ms. There are no latency differences between the responses of both modalities.
We also computed the time course of the theta activity at electrode Cz and found no statistical difference between the theta peak of tactile and painful responses. The peak of the tactile trials was at 293±37 ms of the painful trials at 279±39 ms. Please note that the data were slightly smoothed at subject level in order to prevent double peaks which occurred in some subjects. This smoothing does not affect the general shape of the deflection but provides a better estimate of the latency.

## Slide 2
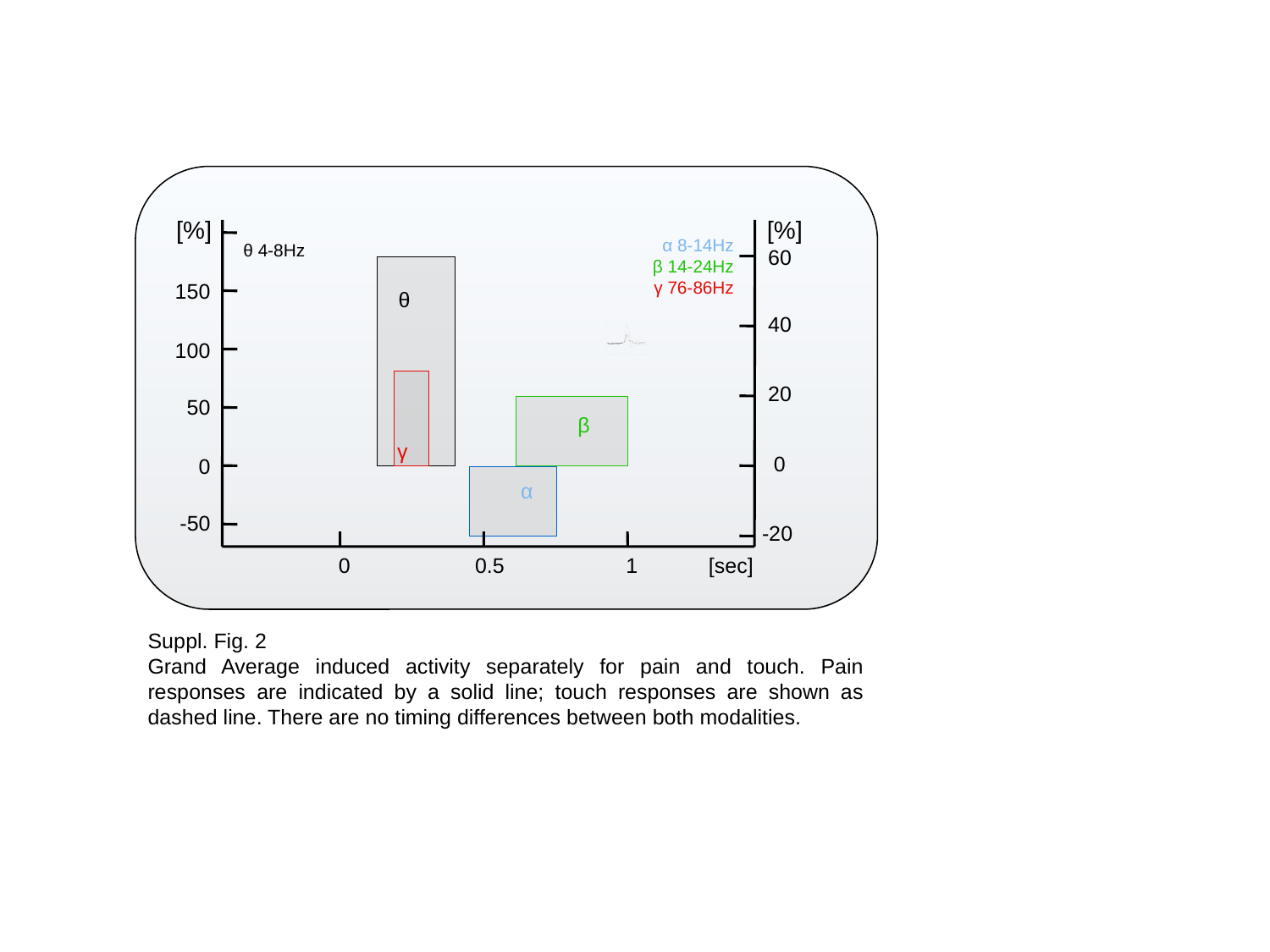

[%]
[%]
α 8-14Hz
β 14-24Hz
γ 76-86Hz
θ 4-8Hz
 60
 40
 20
 0
-20
150
100
50
0
-50
θ
β
γ
α
0 	 0.5	 1 [sec]
Suppl. Fig. 2
Grand Average induced activity separately for pain and touch. Pain responses are indicated by a solid line; touch responses are shown as dashed line. There are no timing differences between both modalities.

## Slide 3
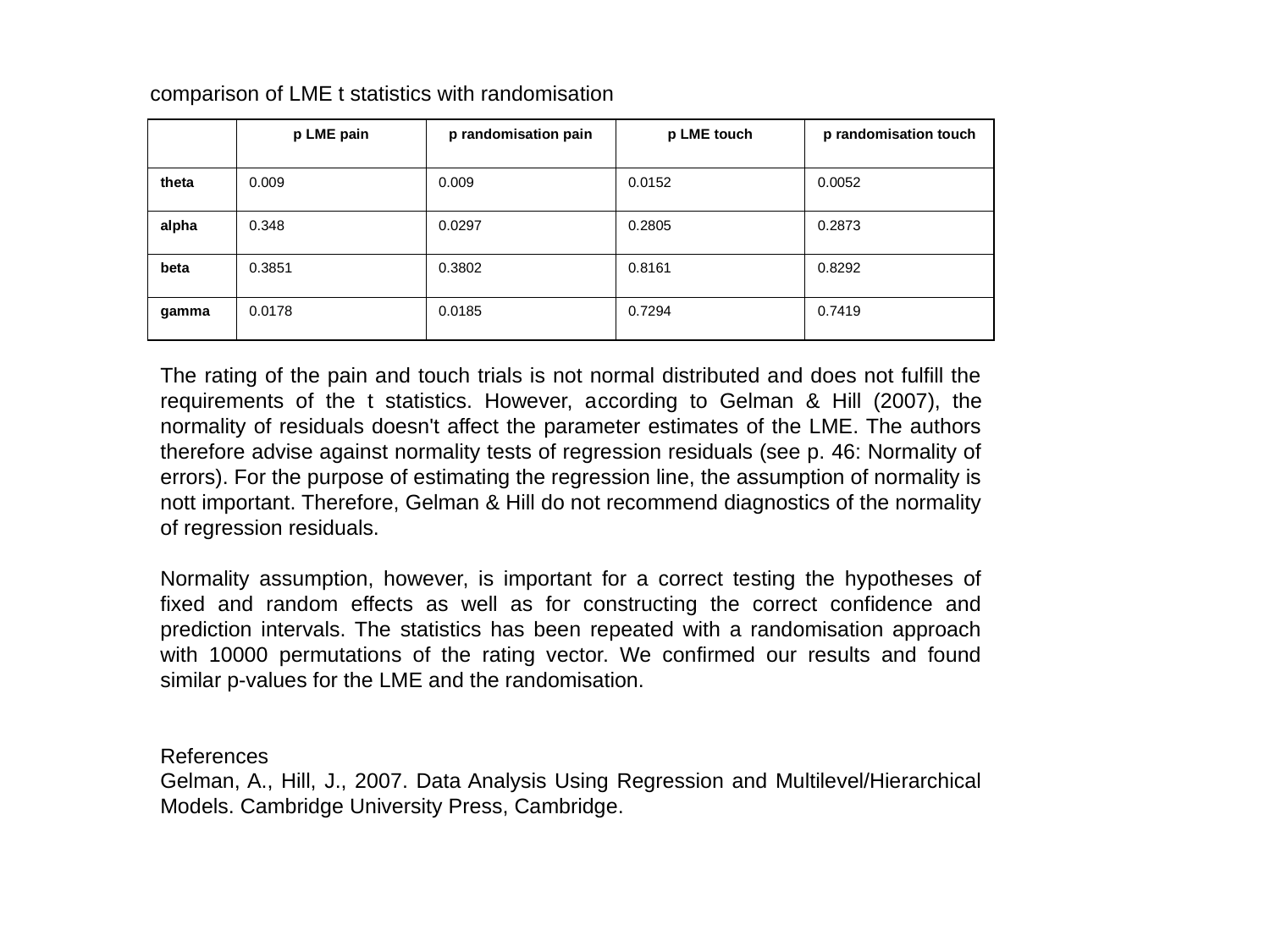

comparison of LME t statistics with randomisation
| | p LME pain | p randomisation pain | p LME touch | p randomisation touch |
| --- | --- | --- | --- | --- |
| theta | 0.009 | 0.009 | 0.0152 | 0.0052 |
| alpha | 0.348 | 0.0297 | 0.2805 | 0.2873 |
| beta | 0.3851 | 0.3802 | 0.8161 | 0.8292 |
| gamma | 0.0178 | 0.0185 | 0.7294 | 0.7419 |
The rating of the pain and touch trials is not normal distributed and does not fulfill the requirements of the t statistics. However, according to Gelman & Hill (2007), the normality of residuals doesn't affect the parameter estimates of the LME. The authors therefore advise against normality tests of regression residuals (see p. 46: Normality of errors). For the purpose of estimating the regression line, the assumption of normality is nott important. Therefore, Gelman & Hill do not recommend diagnostics of the normality of regression residuals.
Normality assumption, however, is important for a correct testing the hypotheses of fixed and random effects as well as for constructing the correct confidence and prediction intervals. The statistics has been repeated with a randomisation approach with 10000 permutations of the rating vector. We confirmed our results and found similar p-values for the LME and the randomisation.
References
Gelman, A., Hill, J., 2007. Data Analysis Using Regression and Multilevel/Hierarchical Models. Cambridge University Press, Cambridge.

## Slide 4
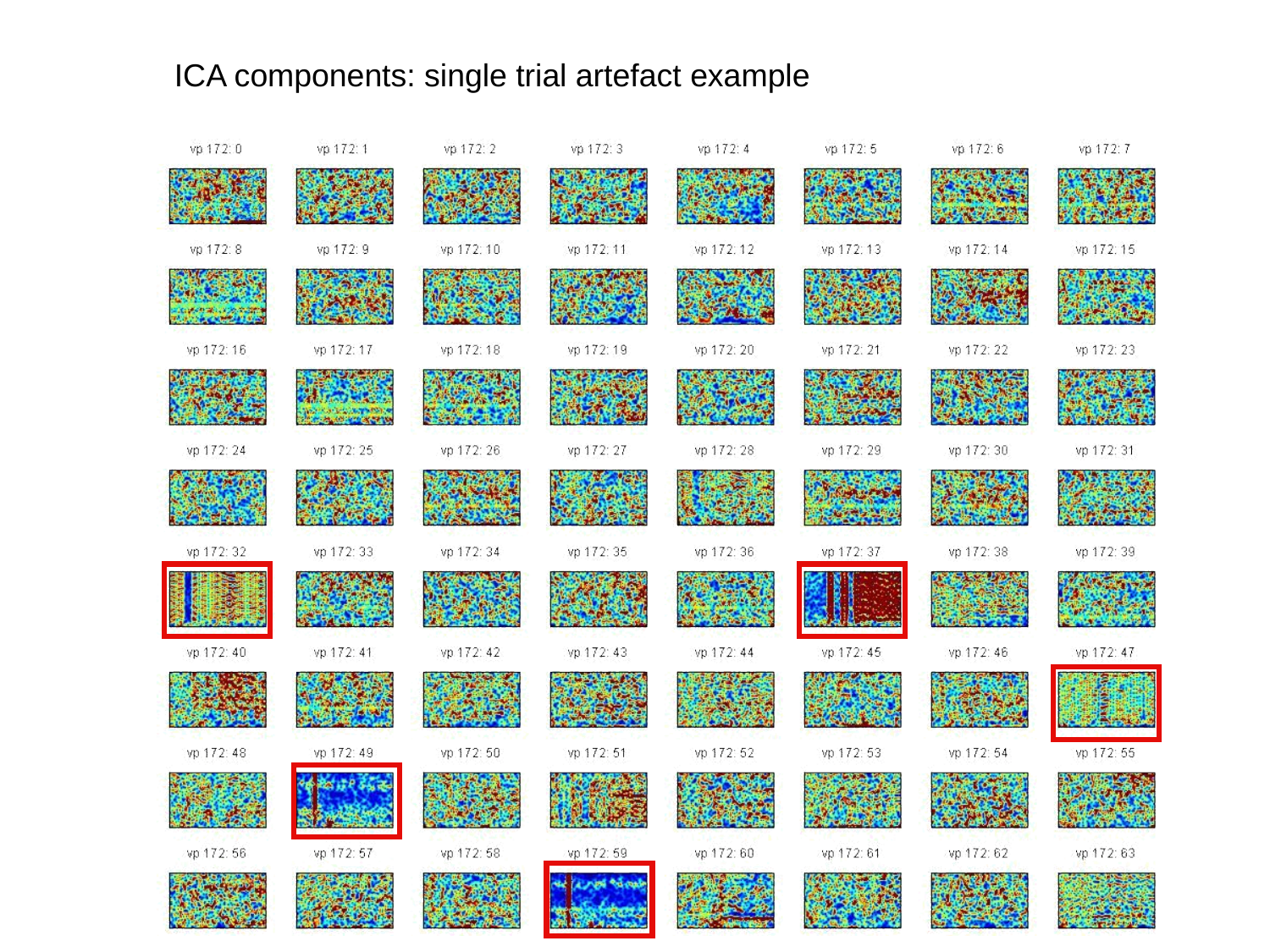

ICA components: single trial artefact example

## Slide 5
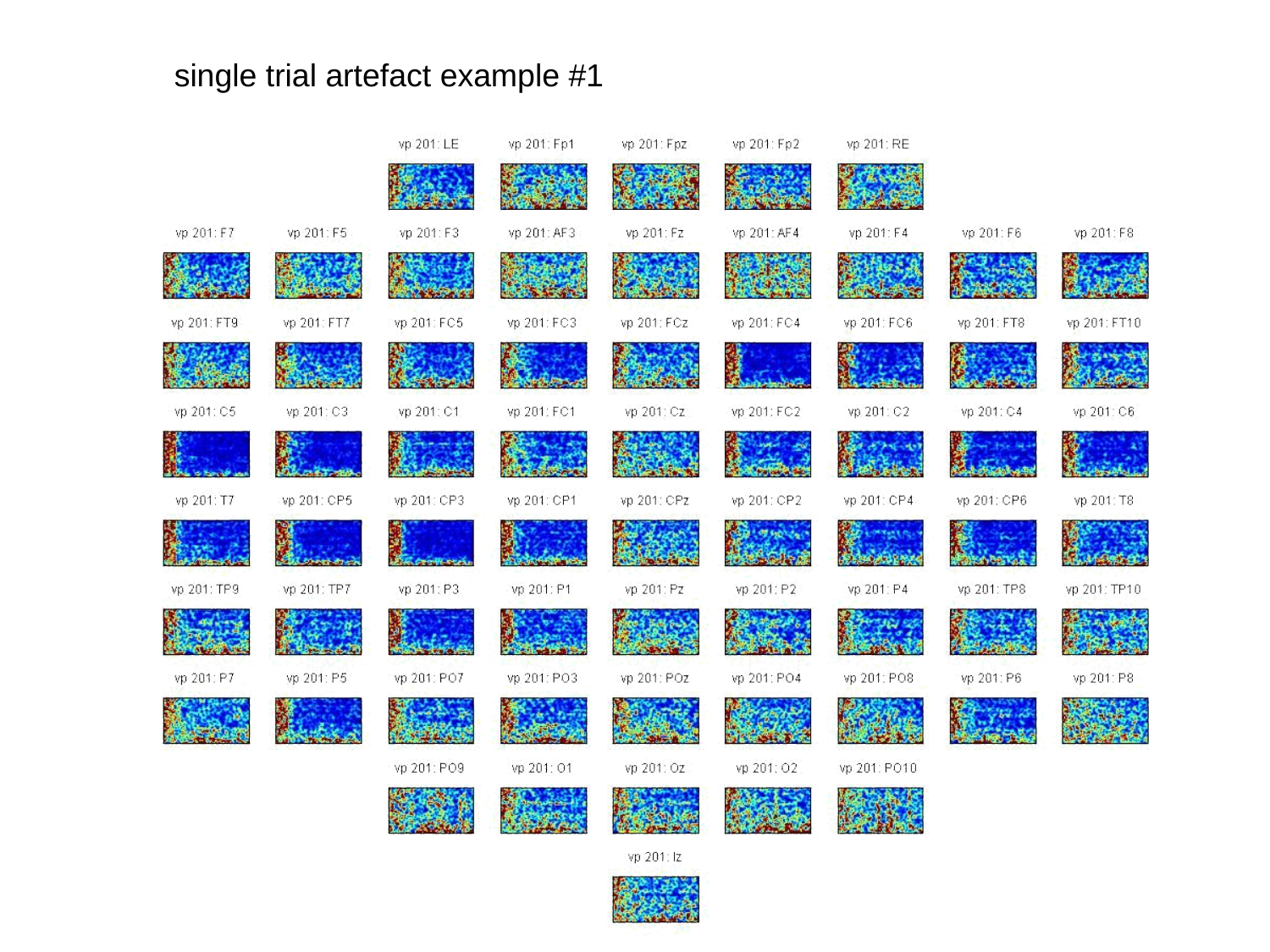

single trial artefact example #1

## Slide 6
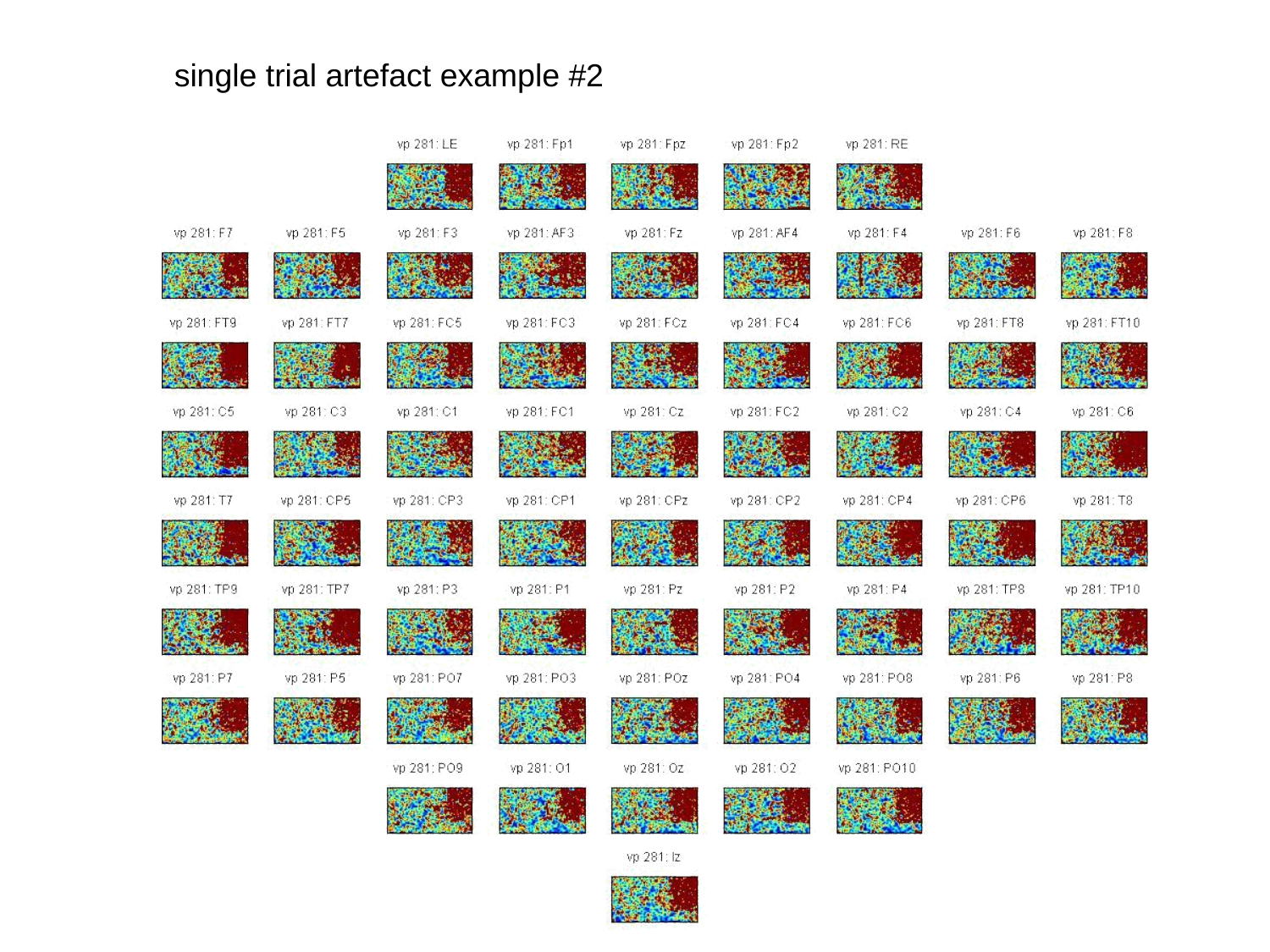

single trial artefact example #2

## Slide 7
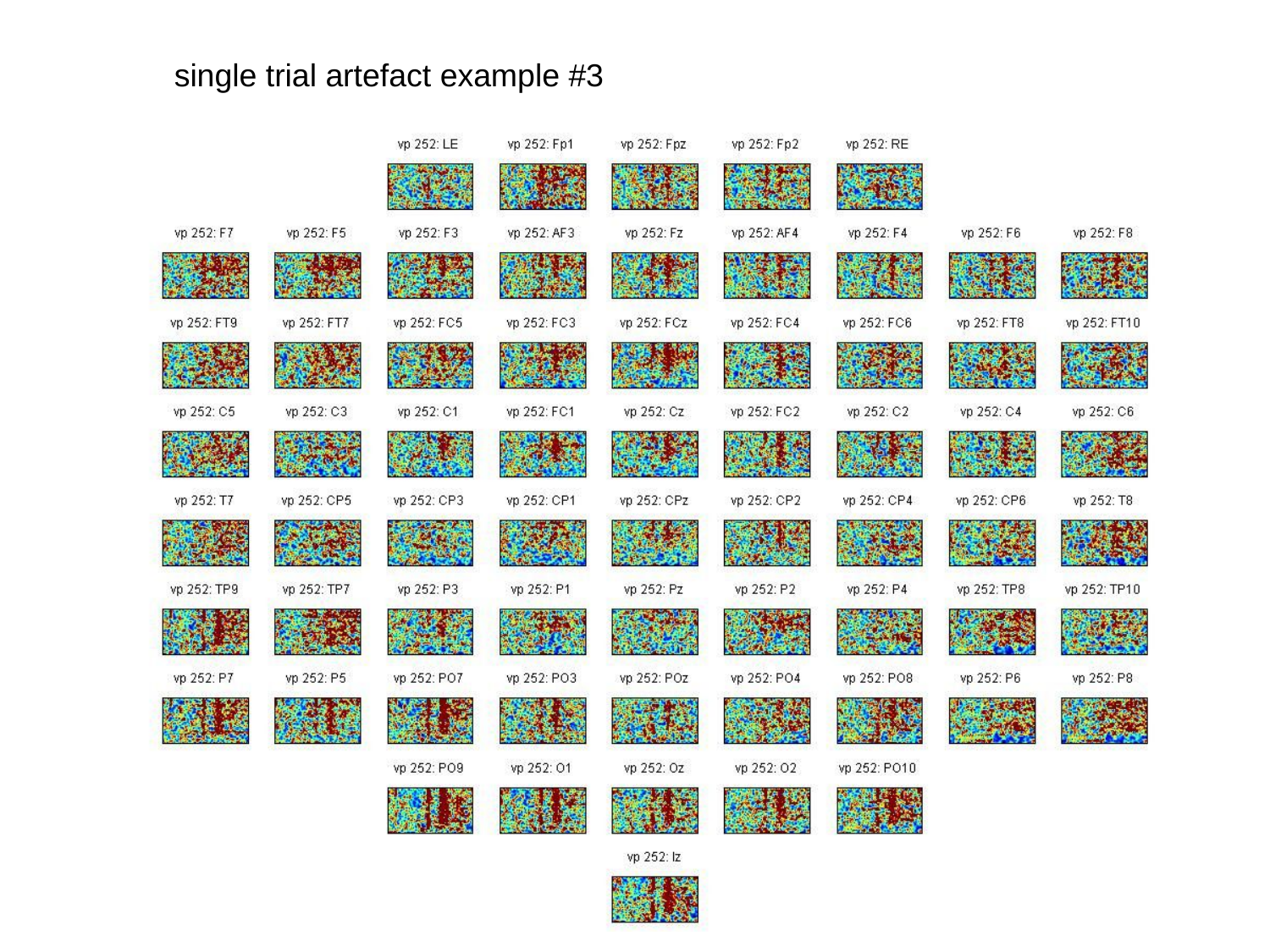

single trial artefact example #3
